# Comparison of Intrathecal Chloroprocaine With Bupivacaine in Short Gynecological Procedures: A Randomized Double-Blind Study

**DOI:** 10.7759/cureus.44187

**Published:** 2023-08-27

**Authors:** Bisman Jeet Kaur Khurana, Sujata Choudhary, Meghna Singhal, Rajesh S Rautela, Rashmi Salhotra, Alpana Singh, Seema Meena

**Affiliations:** 1 Anesthesiology and Intensive Care, Post Graduate Institute of Medical Education and Research, Chandigarh, IND; 2 Anesthesiology, Vardhman Mahavir Medical College and Safdarjung Hospital, New Delhi, IND; 3 Anesthesiology, Maulana Azad Medical College, New Delhi, IND; 4 Anesthesiology, University College of Medical Sciences and Guru Teg Bahadur (GTB) Hospital, New Delhi, IND; 5 Obstetrics and Gynecology, University College of Medical Sciences and Guru Teg Bahadur (GTB) Hospital, New Delhi, IND; 6 Anesthesiology and Critical Care, Max Hospital Patparganj, New Delhi, IND

**Keywords:** gynecology surgery, intrathecal chloroprocaine, 0.25% bupivacaine, spinal anesthesia, day case surgery

## Abstract

Background

Neuraxial anesthesia, compared to general anesthesia, offers better patient comfort, early ambulation, and discharge with excellent post-operative pain relief for short gynecological procedures. Recently chloroprocaine, a short-acting local anesthetic agent became available for intrathecal use. This study aimed to compare intrathecal chloroprocaine with bupivacaine in short gynecological procedures.

Methodology

Consecutive patients undergoing short gynecological procedures, patients belonging to the American Society of Anesthesiology (ASA) I and II, between 18 and 60 years of age, and patients with a height between 150 cm and 180 cm were included in the study. Randomization was done using a computer-generated random number table. Patients were allocated to one of the two study groups. Group B received 4 mL of isobaric bupivacaine (0.25%) 10 mg intrathecal, and Group C received 4 mL of isobaric chloroprocaine (1%) 40 mg intrathecal. The primary outcome criteria were time to ambulation and discharge readiness. The secondary outcome criteria were onset, duration, and intensity of sensory and motor blockade, time to voiding, and any adverse effects.

Results

Patients receiving chloroprocaine had a significantly (p=0.001) faster time (158±31 min) to ambulation compared to bupivacaine (241±23 min). The regression of sensory blockade was substantially faster (p=0.001) with chloroprocaine (60±13 min) than with bupivacaine (94±24 min). Mean time to motor onset was significantly (p=0.05) faster in chloroprocaine (8±3 min) than bupivacaine (12±3 min) group. Significantly faster (p=0.001) recovery of motor blockade was observed with chloroprocaine (130±32 min) than bupivacaine (211±22 min). The time to first voiding was also significantly earlier with stable hemodynamics and no adverse effects in chloroprocaine group.

Conclusion

Intrathecal chloroprocaine may be an attractive alternative and is superior to isobaric bupivacaine as it provides early ambulation and discharge readiness for daycare anesthesia in short gynecological procedures.

## Introduction

Daycare ambulatory gynecological surgeries are routinely performed in tertiary care hospital settings due to their relatively short duration and relatively healthy patient profile. Primary importance is given to patient comfort, efficiency, early recovery, and excellent post-operative pain management. Routinely performed procedures are hysteroscopy, vulvar biopsy, dilation, and curettage. Various techniques like general anesthesia, central neuraxial blockade, intracervical local anesthetic injection, paracervical block, and oral analgesics have been used for intra-operative and post-operative analgesia for early recovery and ambulation for short-duration gynecological procedures.

Intrathecal anesthesia provides complete analgesia and gives the advantage of early-onset, good safety profile, faster recovery, and early ambulation in comparison with general anesthesia [1,2]. Intrathecal anesthesia with short-acting local anesthetic agents can provide adequate muscle relaxation and pain relief during the procedure along with early ambulation and discharge in the post-operative period. At present hyperbaric bupivacaine is used for intrathecal administration but is associated with intense motor blockade and urinary retention in the post-operative period, further delaying ambulation and early discharge.

Chloroprocaine is an amino ester local anesthetic agent with a short half-life. Various clinical trials have shown that 30 mg and 60 mg doses of chloroprocaine provide adequate spinal anesthesia for short-duration procedures lasting 40-90 min [3]. The early onset and faster recovery profile of chloroprocaine make it suitable for short gynecological procedures. Different studies have compared chloroprocaine with bupivacaine; data regarding comparison with isobaric bupivacaine remains limited [4,5]. We conducted a study to compare the effects of intrathecal chloroprocaine with isobaric bupivacaine in patients undergoing short gynecological procedures.

This article was previously presented as an abstract at the 2019 European Society of Regional Anaesthesia and Pain Therapy (ESRA) Annual Conference on September 12, 2019.

## Materials and methods

Study design and participants

The study was a prospective, double-blinded, randomized controlled trial carried out at a tertiary care institute in North India. The trial was registered under the Central Trial Registry of India (#CTRI/2018/01/011267) and approved by the Institutional Ethics Committee of University College of Medical Sciences and Guru Teg Bahadur (GTB) Hospital (#IEC-HR/2017/32/12). Written and informed consent was obtained from each participant before enrolment.

The study was conducted from January 2018 to April 2019 at the University College of Medical Sciences, New Delhi. Consecutive patients undergoing short gynecological procedures (defined as estimated time of procedure between 40 and 60 min) at the institute were evaluated for inclusion in the study. Patients belonging to American Society of Anesthesiology (ASA) I and II, between 18 and 60 years of age, and with a height between 150 cm and 180 cm were included in the study. Patients with a contraindication to subarachnoid block, allergy to drugs used in this study, BMI more than 30 kg/m^2^, history of long-term opioid use, and a history of chronic pain were excluded.

Study procedure

A routine pre-anesthetic assessment was performed on all the patients undergoing short-duration gynecological procedures and the technique of spinal anesthesia was clearly explained to the patients. The patients were prescribed tab alprazolam 0.25 mg the night before and on the morning of surgery.

In the operation theatre, monitoring was carried out for patients using continuous electrocardiography, pulse oximetry, and a non-invasive blood pressure monitor. Under all aseptic precautions, the subarachnoid block was performed via the midline approach at L3-4/L4-5 interspace using a 25G Quincke’s (Medispine Spinal Needle; Selaqui, India: Global Medikit Limited) needle with the patient in a sitting position.

Randomization and Blinding

Patients were randomized to one of the two groups based on a computer-generated table of random numbers. Allocation concealment was done using sequentially numbered sealed opaque envelopes. Group B received 4 mL of isobaric bupivacaine (0.25%) 10 mg intrathecal and Group C received 4 mL of isobaric chloroprocaine (1%) 40 mg intrathecal. The intrathecal drug was prepared by an anesthesiologist not involved in the study. The time of intrathecal drug injection was noted and all the observations were made using this time as "0" min.

Sensory block was assessed by a pinprick method using a 27G hypodermic needle and motor block was assessed using the modified Bromage scale. Readiness for surgery was taken as loss of pinprick sensation at T10 dermatome and complete motor block (modified Bromage scale of <2).

The duration of sensory block was defined as time to two-segment regression of sensory block, duration of motor block was defined as the modified Bromage scale of six. Time to ambulation and time to discharge readiness were taken as primary endpoints. Post Anesthesia Discharge Scoring System (PADSS) was used to determine home readiness. The maximum score was 10 and a score ≥9 was considered fit for discharge [6].

Heart rate, systolic blood pressure, diastolic blood pressure, and mean arterial pressure were recorded during the intra-operative period and every half hour in the post-operative period till complete recovery. Hypotension was defined as a decrease in systolic blood pressure (SBP) below 90 mmHg or a fall in the blood pressure >20% of the baseline value. It was treated with fluids and vasopressors (inj. mephentermine 6 mg IV), as required. Bradycardia was defined as a heart rate of fewer than 50 beats per minute or a decrease in heart rate by 20% of baseline and was treated with 0.6 mg of atropine IV. Patients were given rescue analgesia with intravenous diclofenac 75 mg when Visual Analog Score (VAS) was greater than or equal to three.

The primary outcomes of the study were to compare the time to ambulation and discharge readiness following the administration of the two drugs. The secondary outcomes of the study were to compare time to readiness for surgery, to compare the time of onset and maximum level of sensory block, the time to achieve maximum level and duration of sensory blockade, and to compare the time of onset, intensity, and duration of motor block. Time to voiding, the first requirement for analgesia, and the incidence of adverse effects were also assessed.

Sample size

The sample size was calculated considering the median and range of the time to ambulation as 142.5 min (86-454 min) in the chloroprocaine group and 290 min (190-490 min) in the bupivacaine group [4,7]. To estimate a difference of 148 min in median value a sample size of 10 cases was required in each group at alpha error of 5% and power 90%. However, due to the availability of time, additional patients continued to be recruited for the study. Fifty patients were screened for inclusion in the study, out of which 43 patients fulfilled the criteria and were randomized to the two intervention arms as follows: 21 patients in group B and 22 patients in group C.

Statistical analysis

The data was entered in MS Excel spreadsheet and analysis was done using Statistical Package for Social Sciences (SPSS) version 21.0 (Armonk, NY: IBM Corp.). Categorical variables were presented in number and percentage (%) and continuous variables were presented as mean±SD and median. The normality of data was tested by the Kolmogorov-Smirnov test. Quantitative variables were compared using an independent t-test/Mann-Whitney U test (when the data sets were not normally distributed) between the two groups and paired t-test/Wilcoxon signed-rank test was used for comparison within the group pre- and post-intervention. Repeated measure ANOVA was used to compare hemodynamic variables within the group across follow-up. Qualitative variables were correlated using the chi-square test. A p-value less than 0.05 was considered statistically significant.

## Results

**Figure 1:**
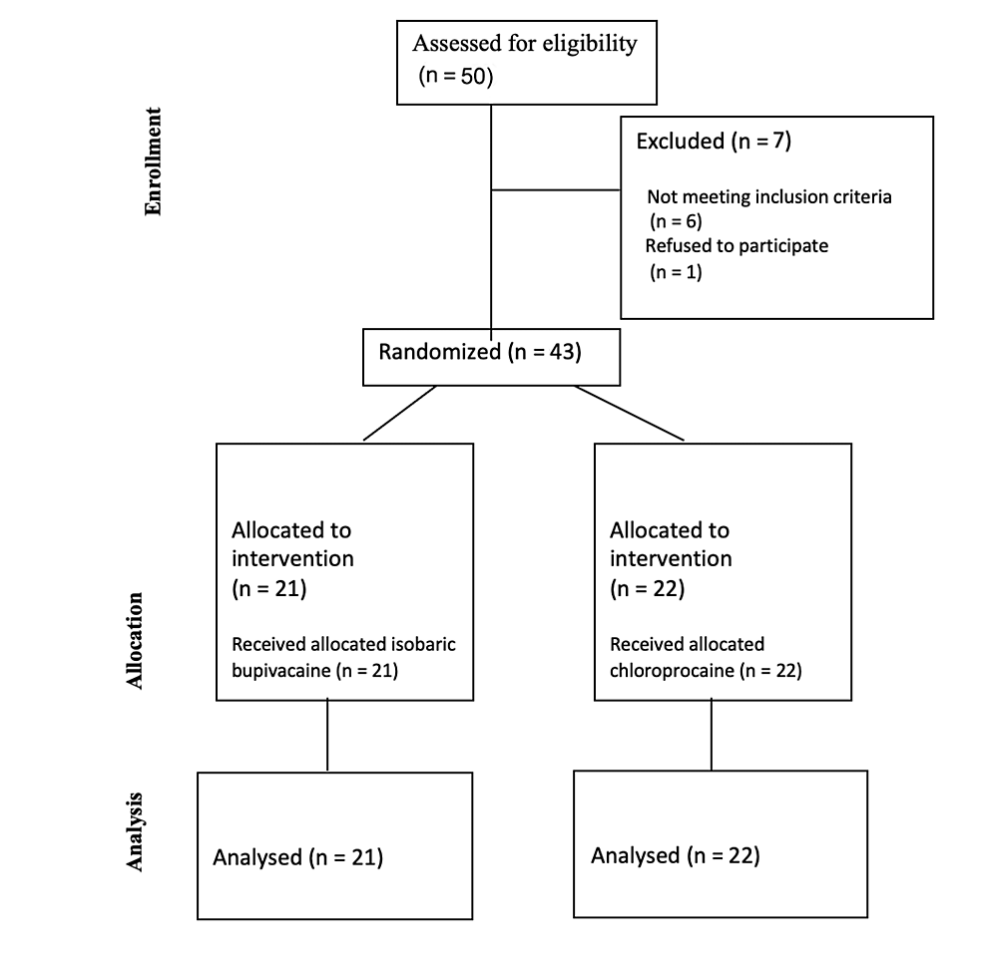
Consolidated Standards of Reporting Trials (CONSORT) flow diagram. n: number of patients

Forty-three patients were randomized and included in the study for analysis (Figure 1). The baseline characteristics of the study population were equally distributed in both groups (Table 1). The most frequently performed procedure was hysteroscopy (79%) followed by marsupialization for Bartholin's cyst (10%) and dilation curettage (7%).

**Table 1:** Demographic profiles of the two groups. n: number of patients

Variables	All patients (n=43)	Chloroprocaine group (n=22)	Bupivacaine group (n=21)	p-Value
Age	42 years (range: 20-68)	42 years (range: 20-63)	45 years (range: 23-68)	0.849
Weight (kg)	59±11.9	57±9.6	60±13.8	0.230
Height (cm)	154±3.9	154±3.1	155±4.6	0.365
Duration of surgery (min)	30±9.4	30±10.0	30±9.0	0.787

The mean time to ambulation and discharge readiness (assessed by PADSS) was achieved significantly earlier in patients receiving chloroprocaine as compared to patients receiving bupivacaine (158±31.4 vs. 240±23.2 min; p=0.000). In addition, patients had a shorter time to maximum motor block (8.5±3.3 vs. 12±3.6 min; p=0.003) and time to motor recovery (129±32.4 vs. 210±22.5 min; p=0.000) in the chloroprocaine group as compared to the bupivacaine group. The time to first voiding was also significantly shorter in patients who received chloroprocaine as compared to patients who received bupivacaine (190±24.6 vs. 285±40.1 min; p=0.000). The different intrathecal block characteristics are highlighted in Table 2.

**Table 2:** Comparison of time to ambulation, sensory block and motor block parameters, and duration of analgesia in two groups. n: number of patients *P-value<0.05 denotes that both groups are significantly different from each other.

Variables	Chloroprocaine group (n=22)	Bupivacaine group (n=21)	p- Value*
Time to ambulation (min)	158±31.4	240±23.2	0.000
Time of onset of sensory block (min)	5±2.4	6±3.9	0.002
Time to maximum sensory block (min)	11±2.6	12±2	0.229
Time to maximum motor block (min)	8.5±3.3	12.2±3.6	0.003
Time to motor recovery (min)	129±32.4	210±22.5	0.000
Time to first voiding (min)	191±24.6	272±40.1	0.000
Duration of analgesia (min)	60±13.9	94±24.3	0.002

**Figure 2:**
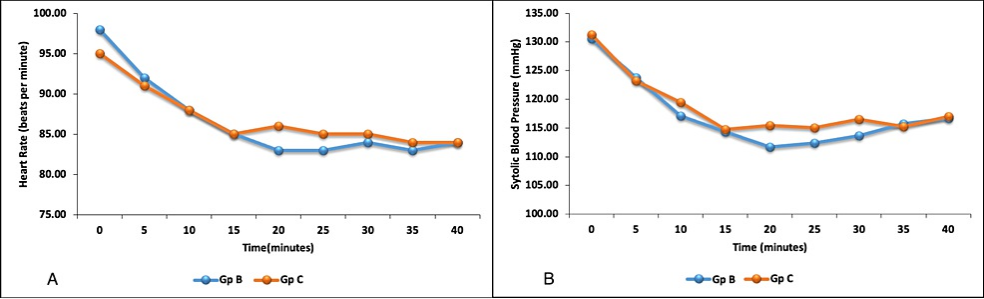
Hemodynamic trend - (A) heart rate and (B) systolic blood pressure of patients during the procedure.

There was no difference in time to the onset of sensory block, time to maximum sensory block, and use of rescue analgesics in the post-operative period in the two groups. Hemodynamic parameters were comparable in the two groups (Figures 2A, 2B). Hypotension was reported in one patient who received bupivacaine which responded to fluid bolus administration. None of the patients in the two groups had adverse effects like shivering, nausea, vomiting, or urinary retention.

## Discussion

Our study compared the time to ambulation and discharge readiness between the commonly used 10 mg of 0.25% plain bupivacaine and the recently introduced 40 mg of 1% chloroprocaine. The study demonstrates that chloroprocaine (158±31.4 min) provides early ambulation and discharge readiness in comparison to plain bupivacaine (240±23.2 min).

The results of this study are in concurrence with the results of a previous study by Camponovo et al. in which the mean duration to ambulation was longer in patients receiving 10 mg bupivacaine (290 min) than in patients receiving 50 mg chloroprocaine (142 min) [5,8,9]. Teunkens et al. also reported early ambulation in patients receiving chloroprocaine (3.2 h) as compared to patients receiving bupivacaine (4.7 h) [10]. In the study by Lacasse et al., early ambulation was reported in patients receiving 40 mg chloroprocaine (255 min) in comparison to patients receiving 7.5 mg bupivacaine (265 min) [4]. The difference in ambulatory time concerning chloroprocaine and bupivacaine can be attributed to the different doses used, drug concentrations used, and different patient profiles studied in the above trials.

In this study, the onset of sensory block observed in the chloroprocaine group (5.14±2.4 min) was comparable to the bupivacaine group (6.86±3.9 min, p=0.002). The results of the present study are similar to previous studies concerning the onset of sensory blockade for chloroprocaine (5-12 min) and bupivacaine (6-12 min) [4,11]. The results of our study are in concurrence with the previous studies, reporting prolonged sensory blockade with bupivacaine (94.0±24.41 min) when compared to chloroprocaine (60±13.96 min, p=0.000), even when different dermatomal levels were defined as endpoints [4,5,10,11]. In the study by Camponovo et al., the duration of sensory blockade (defined as regression to S1 dermatomal level) was longer in patients receiving 10 mg of 0.5% bupivacaine (225 min) than in patients receiving 50 mg of 1% chloroprocaine (105 min) [5]. Similarly, Lacasse et al. reported that the duration of sensory block (defined as regression to L1 dermatomal level) was longer in patients receiving 7.5 mg of 0.75% hyperbaric bupivacaine (75 min) as compared to patients receiving 40 mg of 2% chloroprocaine (50 min) [4]. Teunkens et al. also reported prolonged sensory blockade (return of cold sensation at S5 level was taken as the end point of the sensory blockade) in patients receiving 7.5 mg of 0.5% plain bupivacaine (366 min) than in patients receiving 40 mg of 1% chloroprocaine (126 min) [10].

In the present study, the time taken for maximum motor block (defined as the time to reach modified Bromage scale ≤3) was significantly earlier in patients receiving intrathecal 40 mg of 1% chloroprocaine (8.64±3.3 min) as compared to patients receiving intrathecal 10 mg of 0.25% plain bupivacaine (12.24±3.66 min, p<0.05). The results of the present study are in contrast to the results of the previous studies conducted by Yoos et al., Camponovo et al., and Lacasse et al. that did not find a significant difference in the mean time to achieve maximum motor block, the difference in time to maximum motor blockade concerning chloroprocaine and bupivacaine can be attributed to the different doses used, drug concentrations used and different demographic profiles of the patients studied in the above trials [4,5,11]. 

The duration of the motor block was defined as the mean time taken to reach the modified Bromage scale ≥5 from the time of intrathecal injection. The mean duration of motor blockade was significantly prolonged in patients receiving intrathecal bupivacaine (211.24±22.53 min) as compared to patients receiving intrathecal chloroprocaine (130.32±32.47 min, p=0.02). The results of the present study are in concurrence with the results of the previous studies, where the prolonged duration of motor blockade was reported in the bupivacaine group in comparison to the chloroprocaine group [4,5,11]. The mean duration of motor blockade achieved with chloroprocaine in literature is anywhere between 59 and 100 min, whereas the mean duration of motor blockade with bupivacaine is between 80 and 210 min. This wide range of duration with intrathecal bupivacaine can be attributed to the different patient profiles, different baricity, and different doses of bupivacaine used. The dense motor blockade and prolonged recovery profile of bupivacaine make it a less favorable choice for ambulatory surgeries.

In our study, none of the patients in the two groups had adverse effects like shivering, nausea, vomiting, urinary retention, and headache. No incidence of transient neurological symptoms was observed. Similar results were reported by Camponovo et al. and Yoos et al. where no significant side effects were reported with the two drugs [5,11]. In contrast to the present study, Lacasse et al. reported one case of transient neurological symptoms in both the chloroprocaine and bupivacaine groups [4]. These were females between the age of 50 and 60 years, undergoing trans-obturator tension-free urethral suspension (TVT-O) in the lithotomy position. Lithotomy position and TVT-O are known risk factors for neuropathies hence Lacasse et al. could not confirm the diagnosis of transient neurological symptoms in the two cases [4].

This was a single-center study conducted only in females undergoing short gynecological procedures; hence further evaluation is required in different populations, on larger sample sizes, and in patients undergoing other surgical procedures of shorter duration.

## Conclusions

To conclude, the results of our study indicate that chloroprocaine has a shorter duration of motor and sensory block, less intense motor blockade, and also a shorter time to ambulation and discharge readiness. Intrathecal chloroprocaine may prove to be an attractive alternative and is superior to isobaric bupivacaine as it provides early ambulation and discharge readiness when surgical anesthesia of a shorter duration is required in short gynecological procedures on a daycare basis.
